# Collection and Cryopreservation of Hamster Oocytes and Mouse Embryos

**DOI:** 10.3791/1120

**Published:** 2009-03-27

**Authors:** Nuno Costa-Borges, Sheyla González, Elena Ibáñez, Josep Santaló

**Affiliations:** Unitat Biologia Cellular (Facultat de Biociències),

## Abstract

Embryos and oocytes were first successfully cryopreserved more than 30 years ago, when Whittingham *et al.*^1^ and Wilmut ^2^ separately described that mouse embryos could be frozen and stored at -196 °C and, a few years later, Parkening *et al*. ^3^ reported the birth of live offspring resulting from in vitro fertilization (IVF) of cryopreserved oocytes. Since then, the use of cryopreservation techniques has rapidly spread to become an essential component in the practice of human and animal assisted reproduction and in the conservation of animal genetic resources. Currently, there are two main methods used to cryopreserve oocytes and embryos: slow freezing and vitrification. A wide variety of approaches have been used to try to improve both techniques and millions of animals and thousands of children have been born from cryopreserved embryos. However, important shortcomings associated to cryopreservation still have to be overcome, since ice-crystal formation, solution effects and osmotic shock seem to cause several cryoinjuries in post-thawed oocytes and embryos. Slow freezing with programmable freezers has the advantage of using low concentrations of cryoprotectants, which are usually associated with chemical toxicity and osmotic shock, but their ability to avoid ice-crystal formation at low concentrations is limited. Slow freezing also induces supercooling effects that must be avoided using manual or automatic seeding ^4^. In the vitrification process, high concentrations of cryoprotectants inhibit the formation of ice-crystals and lead to the formation of a glasslike vitrified state in which water is solidified, but not expanded. However, due to the toxicity of cyroprotectants at the concentrations used, oocytes/embryos can only be exposed to the cryoprotectant solution for a very short period of time and in a minimum volume solution, before submerging the samples directly in liquid nitrogen ^5^. In the last decade, vitrification has become more popular because it is a very quick method in which no expensive equipment (programmable freezer) is required. However, slow freezing continues to be the most widely used method for oocyte/embryo cryopreservation. In this video-article we show, step-by-step, how to collect and slowly freeze hamster oocytes with high post-thaw survival rates. The same procedure can also be applied to successfully freeze and thaw mouse embryos at different stages of preimplantation development.

**Figure:**
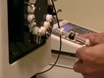


## Protocol

Animal care and procedures were conducted according to the guidelines and regulations approved by the Ethics Committee on Animal and Human Research of the Universitat Autònoma of Barcelona, Spain.

### I. Oocyte collection

Check and select female hamsters (Mesocricetus auratus; 8-12 week’s old) of the Golden Syrian strain for the presence of a thick vaginal discharge (postovulatory discharge).
Induce selected females to superovulate by intraperitoneal injection of 40 IU of PMSG (Pregnant Mare Serum Gonadotropin) followed by 40 IU of hCG (human Chorionic Gonadotropin) 60 hours later.
Euthanize female hamsters 16 hours after administration of hCG in a carbon dioxide chamber.
Isolate oviducts from the females as shown in the video and transfer them to a drop of KSOM-H medium.
Collect oocyte-cumulus complexes by tearing the oviduct ampulla under a stereoscopic microscope in a drop of hyaluronidase (156 U/ml) at 37°C. Oocytes will be freed from cumulus cells in approximately 5 minutes.
Pick up oocytes from hyaluronidase and wash them twice in KSOM-H medium.

For the collection of mouse embryos, female mice (Mus musculus; 6-8 week’s old) are induced to superovulate by intraperitoneal injection of 5 IU of PMSG followed by 5 IU of hCG 48 hours later and mated with male mice. Females are sacrificed by cervical dislocation at 24-26, 44-46 or 54-56 hours after the administration of hCG for obtention of pronuclear, 2-cell or 4-cell stage embryos, respectively. Collection of pronuclear embryos is performed exactly as described for hamster oocytes. Embryos at the 2-cell and 4-cell stages are collected by flushing the oviducts with KSOM-H medium [6].* Note: Following this protocol approximately 20 to 30 oocytes/embryos can be collected from each female.*

### II. Preparation of freezing and thawing solutions

Prepare solution I by diluting 0.57 ml of propilenglycol in 5 ml of KSOM-H (1.5 M of propilenglycol).
Prepare thawing solution by adding 2 ml of KSOM-H to a tube with 0.2052 g sucrose (0.3 M of sucrose).
Prepare freezing solution by adding 2 ml of solution I to a tube with 0.0684 g sucrose (1.5 M of propilenglycol and 0.1 M sucrose).

*Note: Solution I and freezing and thawing solutions must be prepared just before their use. However, the specified amounts of sucrose to be used for the preparation of the freezing and thawing solutions may be weighted in advance and stored in 3 ml-tubes at room temperature for several months.*

### III. Freezing protocol

Transfer oocytes/embryos from the KSOM-H medium to a drop of solution I and incubate for 7-15 minutes at room temperature.
Meanwhile, prepare a 90 mm petri dish to load the straws by placing 90 µl drops of freezing and thawing solutions as it is shown in the video (1 drop of each solution/straw).
Transfer the desired number of oocytes/embryos to the freezing solution drops. *Note: We usually transfer between 5 to 30 oocytes or embryos/drop.*
Attach a straw to a mouth controlled aspiration system and load it following this order: solution I, air, freezing drop with the oocytes, air, thawing drop, air.
Once the straw is loaded, keep aspirating until the first drop introduced (solution I) reaches the polymer at the top end of the straw and seals this end. Thermo-seal the other end of the straw or seal with a plastic plug.
Switch on the programmable freezer and select the freezing program. The freezing program used in this protocol is based on the cooling rates originally published by Lassalle et al. [7]:
  2°C/min from room temperature to -7°C 0°C/min for 10 min to allow for temperature equilibration and induction of seeding (see below)
  0.3°C/min from -7°C to -30°C 0°C/min for 2 min to allow for temperature equilibration
  35°C/min from -30°C to -130°C
Load the straws on the slots of the holder of the programmable freezer, making sure that the part of the straw with the drop of thawing solution is facing outwards. Start the freezing program.
When sample temperature reaches -7°C and the cooling is on hold, perform manual seeding: plunge the forceps into liquid nitrogen, pull the holders slightly out of the freezer to expose the part of the straws containing the drops of thawing solution, and immediately touch this part of the straws with the forceps. Repeat for each holder and each straw in the holder, and then let the freezing program run until the end.
When the program is completed, plunge the holders in liquid nitrogen.
Remove the straws from the holders and transfer them to a labeled gobelet.
Finally, store the gobelet inside a liquid nitrogen tank.

### IV. Thawing protocol

Remove the straw from the liquid nitrogen tank.
Maintain the straw at room temperature for 40 seconds.
Soak the straw in a water bath at 30°C during 40 seconds.
Remove the plug from the straw or cut the thermo-sealed tip.
Empty the straw in a 35-mm petri dish and gently mix the two drops containing the freezing and thawing solutions.
Leave oocytes/embryos in the mixture for 15 minutes at room temperature.
Transfer oocytes/embryos to a fresh drop of KSOM-H for an additional 15 minutes at 37°C.
Oocytes/embryos are now ready for further manipulation.

*Note: During steps 6 and 7 you should see that the thawed oocytes/embryos slowly swell up due to rehydration.*

### V. Representative Results

The use of the cryopreservation protocol reported here results in a survival rate of approximately 95% for hamster oocytes and of >90%, 85% and 80% for mouse B6CBAF1 embryos at the pronuclear, 2-cell and 4-cell stages, respectively. When mouse embryos are cultured in vitro in KSOM after thawing, at least 70 to 80% should reach the blastocyst stage in optimal culture conditions.

It is recommended that you include a control group (straw) of oocytes/embryos in each freezing experiment and that you thaw this group before the rest of the oocytes/embryos are used for further manipulations. If survival rates for the control group are lower than expected, thaw another straw from the same lot. If survival rates for this second group are also low, discard the remaining straws from this freezing lot.

## Discussion

With this protocol hamster oocytes and mouse embryos can be successfully cryopreserved. Once frozen, oocytes/embryos can be stored in liquid nitrogen tanks indefinitely and recovered at any time or place desired. This offers the advantage of having samples almost ready to use whenever needed. On the other hand, this protocol can also be used to generate embryo cryobanks from valuable mouse strains (such as transgenic lines), as an economical and safer alternative to the maintenance of live animal colonies. It is important to note that this protocol is optimized to cryopreserve hamster oocytes and hybrid B6CBAF1 mouse embryos. Differences in post-thaw survival rates among mouse embryos with different genetic backgrounds have been described ^8,9^, thus care should be taken to assess cryopreservation conditions on each species or strain of oocytes/embryos and to establish the appropriate protocol before the generation of oocyte or embryo cryobanks.

Hamster oocytes can be used in human assisted reproduction clinics for the sperm penetration test or for sperm chromosome analysis ^10^, or as practicing material for beginners in intracytoplasmic sperm injection (ICSI). On the other hand, mouse hybrid B6CBAF1 embryos at pronuclear or 2-cell stages can be used in human assisted reproduction centers or in research labs to check the quality of the embryo culture media or the proper functioning of the incubators.
